# *Bacillus anthracis* Edema Factor Substrate Specificity: Evidence for New Modes of Action

**DOI:** 10.3390/toxins4070505

**Published:** 2012-07-06

**Authors:** Martin Göttle, Stefan Dove, Roland Seifert

**Affiliations:** 1 Department of Neurology, Emory University School of Medicine, 6302 Woodruff Memorial Research Building, 101 Woodruff Circle, Atlanta, GA 30322, USA; 2 Department of Medicinal/Pharmaceutical Chemistry II, University of Regensburg, D-93040 Regensburg, Germany; Email: Stefan.dove@chemie.uni-regensburg.de; 3 Institute of Pharmacology, Medical School of Hannover, Carl-Neuberg-Str. 1, D-30625 Hannover, Germany; Email: Seifert.roland@mh-hannover.de

**Keywords:** adenylyl cyclase toxin, anthrax, *Bacillus anthracis*, edema factor, edema toxin

## Abstract

Since the isolation of *Bacillus anthracis* exotoxins in the 1960s, the detrimental activity of edema factor (EF) was considered as adenylyl cyclase activity only. Yet the catalytic site of EF was recently shown to accomplish cyclization of cytidine 5′-triphosphate, uridine 5′-triphosphate and inosine 5′-triphosphate, in addition to adenosine 5′-triphosphate. This review discusses the broad EF substrate specificity and possible implications of intracellular accumulation of cyclic cytidine 3′:5′-monophosphate, cyclic uridine 3′:5′-monophosphate and cyclic inosine 3′:5′-monophosphate on cellular functions vital for host defense. In particular, cAMP-independent mechanisms of action of EF on host cell signaling via protein kinase A, protein kinase G, phosphodiesterases and CNG channels are discussed.

## Abbreviations:

AC: adenylyl cyclase
ACD: adenylyl cyclase domain
ANT: anthraniloyl
EF: edema factor AC toxin from *Bacillus anthracis*
CaM: calmodulin
cAMP: cyclic adenosine 3′:5′-monophosphate
ANTXR: anthrax toxin receptor
CC: cytidylyl cyclase
cCMP: cyclic cytidine 3′:5′-monophosphate
cIMP: cyclic inosine 3′:5′-monophosphate
CMG2: capillary morphogenesis gene 2
cNMP: cyclic nucleoside 3′:5′-monophosphate
CREB: cAMP response element-binding
cUMP: cyclic uridine 3′:5′-monophosphate
cXMP: cyclic xanthosine 3′:5′-monophosphate
IBMX: 3-isobutyl-1-methylxanthine
IC: inosylyl cyclase
LF: lethal factor
mAC: membranous mammalian AC
MAPK: mitogen-activated protein kinase
MAPKK: mitogen-activated protein kinase kinase
MANT: methylanthraniloyl
MRP: multidrug resistance protein
MS: mass spectrometry
NTP: nucleoside 5′-triphosphate
PA: protective antigen
PDE: phosphodiesterase
PKA: cAMP-dependent protein kinase
PKG: cGMP-dependent protein kinase
PMEApp: 9-[2-(phosphonomethoxy)ethyl]adenine diphosphate
PMN: polymorphonuclear leukocytes
sGC: soluble mammalian guanylyl cyclase
TEM: transendothelial migration
TEM8: tumor endothelial marker 8
UC: uridylyl cyclase

## 1. The cAMP Signaling Pathway

**Figure 1:**
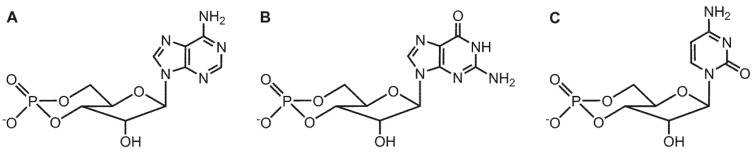
Structural formulas of the second messenger molecules (**A**) cAMP; (**B**) cGMP; (**C**) the potential novel messenger cCMP.

cAMP (cyclic adenosine 3′:5′-monophosphate, Figure 1A) is a ubiquitous intracellular second messenger that regulates numerous cell functions including energy metabolism, cardiac contractility, endocrine function and host immune defense [1,2,3,4]. The formation of cAMP by membranous adenylyl cyclase (mAC) is part of signal transduction cascades including stimulation of integral membrane receptors by hormones, activation of heterotrimeric G proteins and subsequent activation of AC [5,6,7]. Depending on G protein stimulus, AC converts the substrate ATP to cAMP and pyrophosphate. cAMP then exerts biological effects by activation of further signaling proteins, e.g., protein kinase (with subsequent protein phosphorylation steps), cyclic nucleotide-gated ion channels and specific guanine nucleotide exchange factors (GEFs) for small GTP-binding proteins, e.g., exchange protein activated by cAMP (Epac) [8,9,10]. One of the most important intracellular targets of cAMP is protein kinase A (PKA) [11]. cAMP binds to the regulatory subunit of PKA which then dissociates from the catalytic subunit. The catalytic subunit phosphorylates target proteins, changing their function and ultimately resulting in cell type-specific responses. Protein phosphatases dephosphorylate proteins and, thereby, reset the system. As one example demonstrating the importance of the cAMP signaling pathway, binding of hormones to β-adrenoceptors in the heart results in Gα_s_-mediated AC stimulation and hence, elevated cAMP levels increasing cardiac contractility [12,13]. Another endogenous cyclic nucleoside monophosphate (cNMP), cGMP (cyclic guanosine 3′:5′-monophosphate, Figure 1B) constitutes a second messenger that is formed from GTP by guanylyl cyclase and that regulates both endocrine and non-endocrine mechanisms [14,15,16]. Degradation of cAMP and cGMP is accomplished by various isoforms of phosphodiesterases (PDE), a complex superfamily of enzymes consisting of 11 different families [17]. As an alternative mechanism for signal termination, cAMP and cGMP can be transported from the intracellular space into the extracellular space via multidrug-resistance proteins (MRPs) 4 and 5 [18,19,20]. Altogether, the controlled formation of cNMP second messengers in response to receptor stimulation, the occurrence of specific cNMP-mediated biological effects and cNMP degradation as well as cNMP transport represent a sensitive equilibrium essential for the regulation of many cellular processes. Several bacterial toxins very effectively manipulate cAMP signaling in order to impair host defense and to facilitate propagation of the bacterial infection [21,22,23,24,25]. Cholera toxin from the enteropathogenic bacterium *vibrio cholerae* ADP-ribosylates the α-subunit of G_s_ and, thereby, blocks its GTPase activity [26,27]. As a result, Gα_s_ is permanently locked in its active GTP-bound state, resulting in uncontrolled cAMP formation. The massively increased cAMP production leads to profound gastrointestinal secretion, water and electrolyte loss, dehydration and, ultimately, death. *Bacillus anthracis, Bordetella pertussis* and *Pseudomonas aeruginosa* secrete structurally similar adenylyl cyclase exotoxins denoted as edema factor (EF) [28,29,30], CyaA [24,25,31,32,33,34,35] and ExoY [36], respectively.

## 2. The Fatal Synergism of *Bacillus anthracis* Exotoxin Components

**Figure 2:**
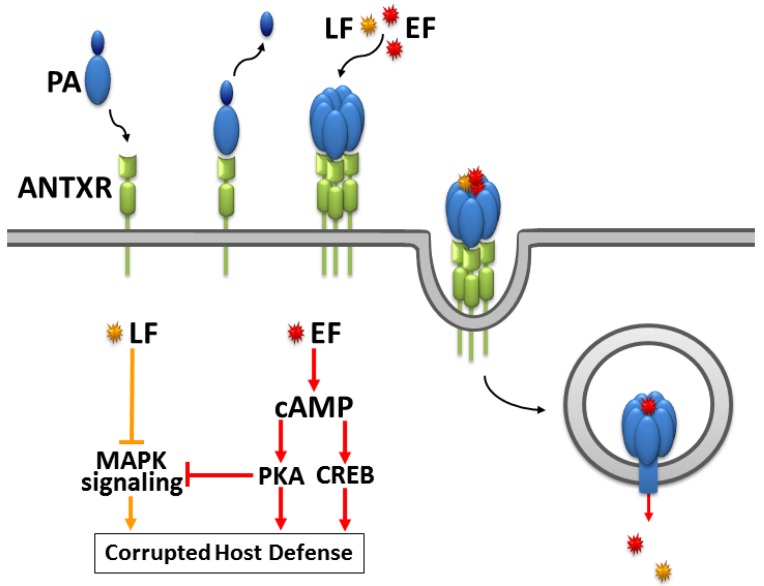
Entry mechanism and synergistic mechanism of action of *Bacillus anthracis* exotoxins according to [29,104]. Upon binding of protective antigen (PA) to anthrax receptors (ANTXR, CMG2 or TEM8), cell-associated furin proteolytic activity cleaves PA into PA_20_ and PA_63_, which self-associates into ring-shaped heptamers binding three molecules of edema factor EF and/or lethal factor LF. The ANTXR-PA-EF/LF complex is endocytosed into an acidic compartment where the high proton concentration mediates conformational rearrangements of the PA prepore allowing EF and LF translocation into the cytosol. In order to achieve a significant impact even at low toxin concentrations, LF and EF synergistically compromise host defense. LF degrades members of the MAPKK family causing inhibited proliferation and cytokine production in T cells, decreased maturation, mobility and cytokine release in macrophages, manipulated cytokine levels in dendritic cells as well as decreased cytokine production and proliferation in B cells. EF produces exceedingly high cAMP concentrations manipulating gene expression via CREB and cell signaling via protein kinase A (PKA). The consequences are decreased cell motility and cytokine production in macrophages, impaired cytokine release from dendritic cells and inhibited chemotaxis in T cells. Interestingly, EF also targets MAPK signaling via PKA. This crosstalk allows the enzymatic activities of EF and LF to synergize in inhibiting MAPK cascades resulting in effectively preventing T cell activation.

Primarily, anthrax is an infection of herbivores caused by pathogenic strains of *Bacillus anthracis*, an aerobic, spore-forming, gram-positive bacterium [37,38,39]. Humans are accidental hosts through the contact with contaminated food, animal products or infected animals. Anthrax disease is divided into four types depending on the mode of entry of the agent: Cutaneous anthrax, gastrointestinal anthrax, inhalational anthrax, and anthrax disease in injection drug users [40,41]. Beginning in the 1960s, *Bacillus anthracis* exotoxins were isolated, and the tripartite nature of anthrax toxin was discovered [42,43]. The three-protein virulence factor includes a 83 kDa protective antigen (PA) [44,45,46,47,48,49,50,51] and two ~90 kDa enzymatic factors, referred to as lethal factor (LF) [52,53,54,55,56,57,58,59,60,61,62,63,64,65,66,67,68,69,70,71,72] and edema factor (EF) [28,73,74,75,76,77,78,79,80,81]. Secreted from the bacteria as nontoxic monomers, the single anthrax toxin components assemble on the cell surface of receptor-bearing host cells to form toxic non-covalent complexes [38,76]. As illustrated in Figure 2, PA specifically binds to the anthrax toxin receptors (ANTXRs) tumor endothelial marker 8 (TEM8) and capillary morphogenesis gene 2 (CMG2) expressed in target cells of the host immune system and assembles into the active holotoxin complex containing a ring-shaped PA oligomer and multiple molecules of LF and EF [82,83,84,85,86,87,88,89]. Anthrax toxin receptors are abundant on endothelial cells of tissues including heart, lung, small intestine, spleen liver, kidney, skeletal muscle, and skin [90,91]. Their physiological roles are still discussed. Recent research points to a role of CMG2 in angiogenesis [92]. CMG2 and TEM8 share similarities with integrins, e.g., a recently resolved TEM8 crystal structure shows typical integrin folds [93]. After receptor binding, a cell-associated furin protease cleaves PA into two fragments [94]. While the smaller 20 kDa fragment PA_20_ dissociates, the larger 63 kDa C-terminal receptor-bound fragment PA_63_ self-associates into ring-shaped heptamers [76,95,96]. The heptamer binds three molecules of EF and/or LF and is endocytosed and trafficked to an acidic intracellular compartment [47,76,97,98,99,100,101]. There, the low pH induces conformational changes in the heptameric PA_63_ moiety allowing it to form a membrane-spanning pore and translocate bound EF and/or LF across the membrane into the cytosol [44,76,102,103].

As demonstrated by electrophysiological measurements, EF and LF translocation is initiated by the interaction of the unstructured N-terminus of the enzyme moieties with a structure formed by the Phe427 residues within the lumen of the oligomeric PA pore [105]. Electron microscopy studies have shown the structure of the heptameric PA channel consisting of a wider, 70 Å long, cap-shaped part probably containing the LF/EF binding sites and a thinner, 100 Å long, stem-shaped part spanning membrane lipid bilayers [106,107]. Moreover, a switch within the CMG2 receptor interacting with two domains of PA determines structural rearrangements within the heptameric PA prepore that are required for pore conversion to an acidic endosomal compartment [108]. The transformation from prepore to membrane-spanning pore requires substantial conformational changes of the PA oligomer [44]. Several models for EF and LF unfolding and translocation through the PA pore have been established supporting active PA-mediated unfolding as well as ∆pH-driven translocation [46,82,109,110]. PA forms mixtures of two noninterconverting oligomers, a homoheptamer and a homooctamer, which have unique properties [45,48,49,50]. LF complexes containing octameric PA possess enhanced stability and channel forming activity, thus higher macrophage cytotoxicity relative to those containing heptameric PA [48]. Depending on receptor levels, PA can lead to macrophage cell death via apoptosis even in the absence of the catalytic subunits EF and LF [51].

LF is a Zn^2+^-dependent metalloprotease cleaving members of the mitogen-activated protein kinase kinase family which is essential for signaling in innate and adaptive immune cells (Figure 2) [71,84,111]. In macrophages, LF inhibits activation, cell motility, chemotaxis, maturation, expression of pro-inflammatory cytokines [29,69,112,113] and induces apoptosis [53,56,62]. In endothelial cells, LF-induced apoptosis has been described, too [114]. In dendritic cells, LF impairs cytokine release and maturation and may induce apoptosis as well [29,52,112]. LF suppresses dendritic cell costimulatory functions inhibiting essential cross-talk between innate and adaptive immune responses [54]. In T and B lymphocytes, LF blocks antigen receptor-dependent proliferation, cytoskeletal rearrangement, cell migration, chemotaxis, cytokine production and Ig production [29,54,104,112,113]. Moreover, in mouse lung, LF modulates the expression of genes regulating vascular permeability and immune specific genes, e.g., transcripts encoding neutrophil chemoattractants, up-regulation of lymphoid genes and down-regulation of myeloid genes [60]. A major role of LF was found for the pathogenesis of anthrax meningitis [61].

**Figure 3:**
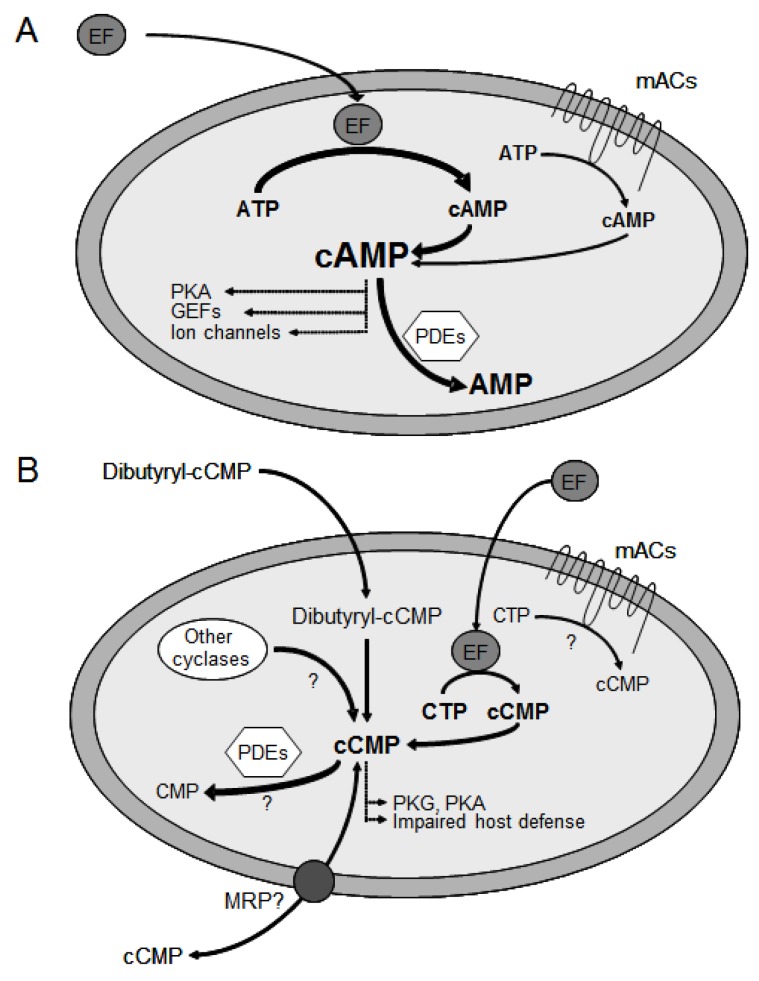
Manipulation of host defense by EF adenylyl cyclase activity (A) and cytidylyl cyclase activity (B). Compared to mammalian membranous ACs (mACs), EF possesses a much higher specific adenylyl cyclase (AC) activity. The excessive cAMP accumulation activates cAMP-dependent protein kinase (PKA), guanine nucleotide exchange factors (GEFs) and ion channels. As a result, host defense is compromised. EF cytidylyl cyclase activity may effect further impairment of immune response by causing intracellular cCMP accumulation. cNMPs are degraded by phosphodiesterases (PDEs). The role of endogenous cCMP as a messenger molecule, the existence of cCMP forming signaling enzymes, PDEs degrading cCMP as well as cCMP transport by multidrug resistance proteins (MRPs) are unknown.

EF is an AC toxin that is inactive outside the host cell. Upon interaction of EF with calmodulin (CaM), an endogenous cytosolic calcium sensor protein, EF undergoes substantial conformational changes, ultimately resulting in exceedingly high catalytic AC activity [28,115]. In fact, the enzymatic activity of the EF-CaM complex exceeds the activity of mACs by several orders of magnitude (Figure 3A). Fluorescence videomicroscopy imaging studies in live cells provided the time course of EF catalytic activity and showed that EF enters the cytosol from late endosomes. Moreover, EF showed perinuclear localization generating cAMP concentration gradients decreasing from the nucleus to the cell periphery [77,116]. EF is lethal to mice, causing multiple tissue damage and cardiovascular malfunction [84,117,118]. A strain of anthrax with a defective EF gene caused 100-fold reduced lethality in mice pointing to major importance of EF in the pathogenesis of anthrax [75]. In a rabbit model of inhalational anthrax, absence of PA resulted in complete avirulence, while the presence of either EF or LF resulted in lethality [119]. The flooding of the host cell with cAMP and the LF-induced inhibition of the MAPK pathway compromise host defense [1]. In particular, EF causes impaired phagocyte function [120,121,122], reduced phagocytic abilities of monocytes [123] and manipulated cytokine secretion in dendritic cells [124]. EF significantly impairs human neutrophil chemokinesis, chemotaxis, actin assembly and polarization [80]. Additionally, inhibition of human neutrophil NADPH oxidase activity was observed [125]. EF-induced deregulation of the PKA system results in impairment of macrophage functions, e.g., inhibited cytokine release, cytoskeletal remodeling and motility as well as reduced phagocytosis [29,81,126]. Moreover, EF activity modulates the expression of macrophage genes responsible for key cellular functions including immune response, inflammation, cell signaling and transcription regulation [78]. The cytotoxic effects of EF can result in macrophage cell death [127]. In B cells, EF modulates cytokine production, inhibits migration and induces apoptosis [78]. In T cells, EF impairs activation, proliferation, cytokine release and chemotaxis [29,128]. In addition, EF affects the Th1/Th2 balance by potently promoting Th2 cell differentiation [30]. Interestingly, a biphasic modulation of PKA- and CREB-dependent signaling by EF has been found. The first intoxication phase consists of EF induced PKA-dependent signaling and CREB phosphorylation as well as activation of gene transcription. Due to negative feedback mechanisms in the second phase, CREB phosphorylation is impaired and therefore T cells are not able to respond to stimuli involving CREB [129].

Most importantly, EF and LF synergize in their action against host immunity [124,128,130] and deletion of either the EF or LF gene leads to reduced virulence of anthrax bacteria [74,75,117]. 

In T cells, EF-induced PKA activity inhibits T cell antigen receptor signaling via MAPK at multiple points of attack, thus potentiating the impact of LF on MAPKKs (Figure 2). As a result from this crosstalk between EF and LF activities, T cell chemotaxis, proliferation and cytokine release are impeded severely even at low toxin concentrations [29,104,112]. Moreover, a cumulative effect of EF and LF activities on inhibition of cytokine release (e.g., TNFα) has been shown in dendritic cells [29,124]. Clinically, the LF-associated peripheral vascular function and myocardial depressant effect synergize with the peripheral vascular effects of EF. Therefore EF and LF may contribute in additive fashion to anthrax-associated shock [131]. Bioluminescence imaging studies coupled with histology demonstrated a central role of EF in virulence in guinea pigs and during inhalational infection in mice. Interestingly, simultaneous production of EF and LF during infection resulted in a specific temporal pattern of histological lesions in the spleen, with early LF-associated lesions, followed later by lesions typical of EF [132].

Since the late 1800s, impressive progress in the development of anthrax vaccines has been made [133,134,135,136]. Particular importance is attributed to the development of therapeutic antibodies directed against the individual toxin factors, against PA [137,138,139,140,141], against EF [142,143,144], against LF [139,144,145] or even bifunctionally directed against EF and LF aiming at toxin crosslinking [146].

Moreover, peptide inhibitors were developed preventing LF and EF binding to PA [147] and inhibitors of calmodulin-induced activation of EF were identified applying allostery concepts [148]. Future efforts will focus on further investigating the molecular mechanisms of action of LF and EF in order to explore these key virulence factors in anthrax pathogenesis as promising pharmacological targets for future treatment options.

## 3. Substrate-Specificity of EF: cCMP, cUMP and cIMP as Potential New Second Messengers

Anthrax infections can effectively be treated with antibiotics. However, in the event of infections with antibiotic-resistant *Bacillus anthracis* strains as well as in toxinemia, further treatment options involving potent and selective EF inhibitors are needed to prevent suppression of immune responses. Various classes of EF inhibitors have been developed including so-called “P”-site agonists (non-competitive substrate analogs) [149,150,151], PMEApp (9-[2-(phosphonomethoxy)ethyl]adenine diphosphate, the active metabolite of the hepatitis B drug adefovir dipivoxil) [152], ethyl 5-aminopyrazolo[1,5-α]quinazoline-3-carboxylate [153], 4-[4-(4-nitrophenyl)-thiazolylamino]-benzene-sulfonamide [154] and 2′(3′)-*O*-(*N*-methylanthraniloyl)-(MANT)-substituted nucleotides [155,156,157]. Studies investigating ANT- and MANT-nucleotides as competitive substrate analogous EF inhibitors were conducted by replacing the 2′- and 3′-*O*-ribosyl position of ATP—the natural EF substrate—with substituted ANT- and MANT-groups and by exchanging the adenine base for various bases including the purines hypoxanthine and guanine as well as the pyrimidines cytosine and uracil. Interestingly, cytosine substituted MANT-nucleotides were found to be particularly potent EF inhibitors (Table 1), and moreover, even the unsubstituted nucleoside triphosphate CTP inhibited EF adenylyl cyclase activity [158,159]. 

**Table 1:** Structure-activity relationships (K_i_ values) for the inhibition of the catalytic activities of recombinant mammalian ACs 1, 2, 5 and the bacterial AC toxin EF by selected MANT-nucleotides. Activities of ACs 1, 2 and 5, as well as CaM-activated EF were determined in the presence of MANT-nucleotides at increasing concentrations and in the presence of 5 mM MnCl_2_. K_i_ values were calculated by non-linear regression. K_i_ values are given in nM and are the mean values of 4 to 5 independent experiments performed in triplicates with at least two different membrane preparations (for mACs). Data were taken from [155,156,157,158,159,160]. For the sake of clarity of the table, SD values are not shown in this summary table. For this information, the reader is referred to the original papers cited above.

Inhibitor	AC 1 (nM)	AC 2 (nM)	AC 5 (nM)	EF (nM)
MANT-ATP	150	330	100	580
MANT-ITP	2.8	14	1.2	4,100
MANT-GTP	90	620	55	2,500
MANT-CTP	150	690	150	100
MANT-UTP	46	460	32	3,700

**Figure 4:**
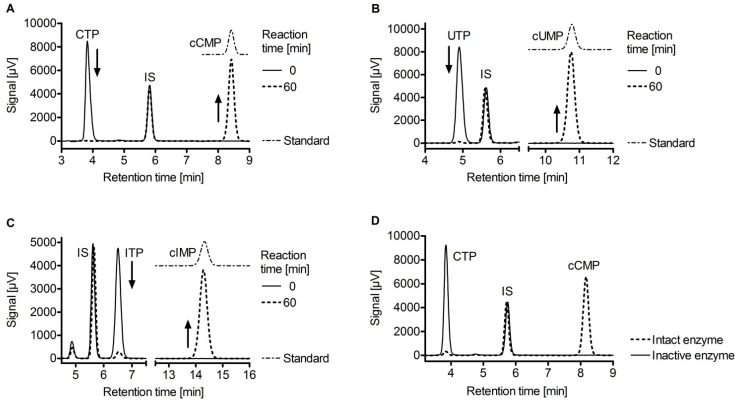
HPLC chromatograms of reaction mixtures consisting of EF and the substrates CTP (20 nM EF and 20 nM CaM) **(A);** UTP (120 nM EF and 120 nM CaM) **(B);** ITP (300 nM EF and 300 nM CaM) **(C).** Samples were withdrawn at indicated reaction times. Chromatograms of standard substances were moved vertically in order to prevent overlapping of the lines. **(D)** Chromatograms with substrate CTP after 60 min reaction time for active and heat-inactivated enzyme. IS, internal standard. Data were taken from [163].

The surprisingly high affinities of CTP and MANT-CTP for EF raised the question if CTP could also serve as a substrate for EF. The analysis of this question is of broader biological importance. Specifically, the physiological existence of cCMP (Figure 1C) in mammalian cells and the potential role of cCMP as a novel second messenger had been claimed already in the 1970s, but due to substantial technical problems including lack of specific antibodies, insufficiently accurate mass spectrometry detection methods and artifacts in the chromatographic separation of cytidine nucleotides, the issue has remained controversial [161,162]. Accordingly, very little research had been conducted in this field during the past two decades. Taking advantage of the high purity of recombinant EF and by modifying *in vitro* enzymological methods based on radioactively labeled substrates as well as by establishing highly sensitive HPLC-MS/MS analytical methods, the catalytic activities of EF on CTP, UTP and ITP were investigated and, as shown in Figure 4, EF was unambiguously found to produce cCMP, cUMP and cIMP besides cAMP [163]. The efficiencies of enzymatic conversion decrease in the order ATP > CTP > UTP > ITP (Table 2). The k_cat_ value of cCMP formation is ~100-fold lower than that of cAMP formation, but as EF enzymatic activities are exceedingly high as compared to endogenous mammalian host cell adenylyl cyclase activities, the rate of cCMP formation is considerable. *In vitro* enzyme activity experiments showed complete conversion of 100 µM CTP, UTP and ITP within 60 min when EF was applied at concentrations of 20 nM, 120 nM and 500 nM, respectively [163]. Moreover, the K_i_ values of various (M)ANT-nucleotides inhibiting EF adenylyl cyclase (AC) activity and cytidylyl cyclase (CC) activity are very similar, corroborating the notion that the AC and CC activities of EF reside in one and the same catalytic site.

**Table 2:** Enzyme kinetics of EF AC, CC and UC activities in the presence of Mg^2+^ or Mn^2+^ nucleotidyl cyclase activity of CaM-activated EF was determined with radiometric enzyme kinetics assays. The toxin was incubated with various substrate concentrations in the presence of either 5 mM Mg^2+^ or 5 mM Mn^2+^. Apparent K_m_ and k_cat_ values were obtained by non-linear regression analysis of substrate-saturation experiments and are the means ± SD of 3 to 4 independent experiments performed in duplicates. Saturation curves were analyzed by non-linear regression. Data were taken from [163].

Enzyme	NC activity	Me^2+^	K_m_ [µM]	k_cat_ [s^-1^]
EF	AC	Mn^2+^	35.3 ± 3.7	501.5 ± 55.9
		Mg^2+^	175.8 ± 29.9	684.2 ± 272.5
	CC	Mn^2+^	12.5 ± 3.4	8.8 ± 1.4
		Mg^2+^	419.7 ± 115.1	7.2 ± 3.1
	UC	Mn^2+^	134.5 ± 23.5	2.3 ± 0.2

These unexpected and newly discovered enzymatic activities of EF raise many questions regarding the mechanism of action of this toxin in host cells. According to the current paradigm [28], EF is a highly active AC toxin that excessively activates endogenous signal transduction pathways in host cells usually regulated by mammalian ACs [3]. Molecular targets of toxin-produced cAMP are cAMP-dependent protein kinase (PKA) [11], cyclic nucleotide-gated ion channels [164,165,166,167] and guanine nucleotide exchange factors, e.g., exchange protein activated by cAMP (Epac) [8,9,10]. Consequently, host defense is compromised. However, there is already evidence in the literature that the effects of EF in intact cells are not necessarily mediated by cAMP:

Voth *et al.* documented the lack of a linear correlation between EF-induced intracellular cAMP accumulation and cell death by investigating the impact of EF on zebrafish Pac2 embryonic fibroblasts, RAW 264.7 and IC-21 murine macrophage-like cells, NIH/3T3 murine fibroblasts as well as CHO cells [127]. Depending on the type of cell analyzed, EF intoxication caused cell death in presence of low levels of cAMP, and, conversely, cell survival was observed in cells in which high levels of cAMP were found following treatment with EF. The authors provide several possible explanations for EF´s cell specific cytotoxicity including that EF “may possess a yet undiscovered activity that contributes to cell death.”

When 11 bioterrorism-related cases of pulmonary anthrax were described in 2001, lung tissues from these patients were found to contain only few intraalveolar inflammatory infiltrates. The pleural fluid of several patients showed decreased numbers of polymorphonuclear leukocytes (PMNs) essential for host defense against bacterial infection [168,169,170,171]. Similarly, the pulmonary interstitium of African Green Monkeys exposed to anthrax spores showed few PMNs only [172]. These combined data suggest an inhibited delivery of circulating PMNs to extravascular sites of infection. Agents that increase intracellular cAMP or behave as cAMP analogs in endothelial cells (EC) are known to enhance EC-EC adhesion, tightening the paracellular pathway, promoting barrier integrity and impairing PMN passage [173,174,175]. Therefore, Nguyen *et al.* have tested the hypothesis if transendothelial migration (TEM) of PMNs is suppressed by EF [171]. Interestingly, it was found that—through its action on ECs—EF decreased IL-8-stimulated TEM of PMNs by ~60%, but moreover, it was even found that the effect of EF on IL-8 driven TEM of PMNs is independent of the PKA pathway. In a time- and dose-dependent manner, EF increased PKA activity in EC and induced phosphorylation of cAMP response element-binding protein (CREB), a direct PKA substrate. Two structurally dissimilar PKA inhibitors, H-89 (N-[2-[[3-(4-bromophenyl)-2-propenyl]amino]ethyl]-5-isoquinolinesulfonamide) and KT-5720, were then tested for their ability to counteract the EF effect on TEM. H-89 and KT-5720 both blocked EF-induced increments in PKA activity and diminished EF-induced CREB phosphorylation. However, both inhibitors had no impact on the EF-induced reduction of TEM. Therefore, the effect of EF on TEM is unlikely to be mediated by the cAMP-PKA pathway. Moreover, forskolin (10 μM) and IBMX (3-isobutyl-1-methylxanthine, 1 mM) were used to increase cAMP levels and PKA-mediated phosphorylation of CREB in ECs. However, neither forskolin nor IBMX could reconstitute the EF effect on IL-8 driven TEM of PMNs. Although forskolin and IBMX each upregulated PKA activity comparable to that seen after EF treatment, none could decrease TEM. Again, these combined data do not support a cAMP/PKA-dependent mechanism through which EF inhibits TEM of PMNs [171]. Such dissociations between EF-induced cAMP accumulation and EF-induced cellular effects lend support to the hypothesis that other second messenger molecules, *i.e.*, cCMP, cUMP and cIMP, might contribute to EF actions. Thus, in future studies, the EF-induced intracellular formation of various cNMPs depending on time, cellular compartment and cell type as well as the corresponding detrimental effects on cell function remain to be investigated differentially.

## 4. EF Structure and Nucleotide Binding Modes

**Figure 5:**
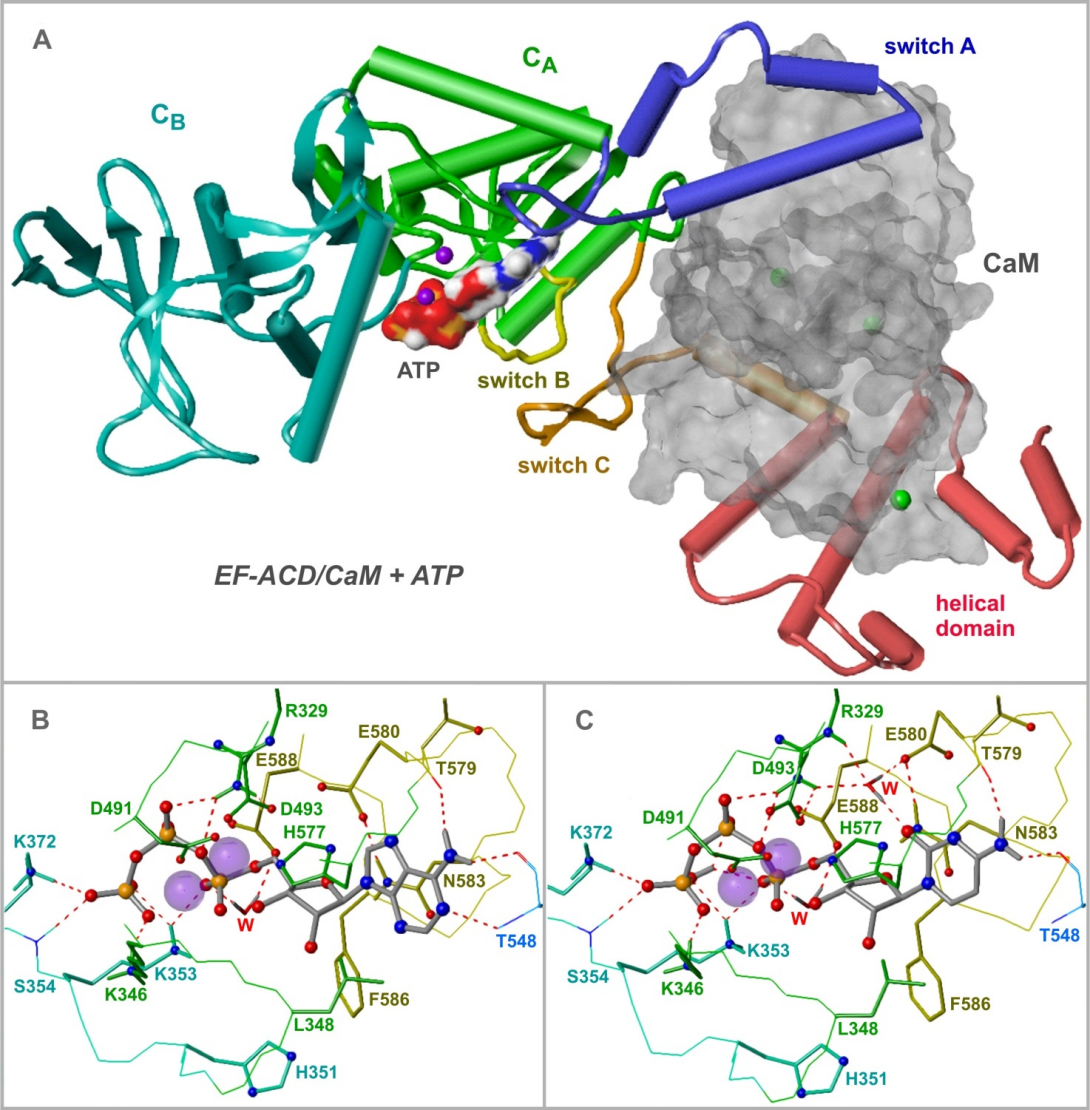
Structure of EF and interactions with ATP and CTP. Models are based on the PDB structure 1xfv [177]. If not otherwise indicated, atoms are colored as follows: C and some essential H of ligands—grey, O—red, N—blue, P—orange, Mg^2+^—magenta, Ca^2+^—green. **(A)** Domain organization of EF, adapted from [178]; helices are drawn as cylinders, β-sheets as arrow ribbons, ATP and CaM (uniformly grey) as Connolly surfaces. **(B, C)** Detailed interactions of ATP (**B**) and CTP **(C)** with EF; the nucleotides and the side chains of amino acids within a sphere of ~3 Å around the ligands and Mg^2+^ ions (space fill) are drawn as sticks, heteroatoms as balls, backbone traces as lines; colors of C, H atoms and backbone traces of EF correspond to the domain: C_A_—green, C_B_—greenblue, switch A—blue, switch B—yellow; w—suggested water molecule; dashed red lines—hydrogen bonds. For details of model generation with the software SYBYL 7.3 (Tripos, L.P., St. Louis, MO, USA), see [163].

EF consists of an N-terminal protective antigen binding domain and an adenylyl cyclase domain (ACD, Figure 5A) comprising two large globular subdomains, C_A_ (D294–N349, A490–K622) and C_B_ (V350–T489). Three flexible "switches" A, B, and C change their conformations upon CaM binding. Whereas switch A (T502-N551) and B (G578-N591) are segments of C_A_, switch C (D623-T659) links C_A_ to a C-terminal helical subdomain (S660-K800) which together with switch A and C provides a large binding site for CaM [176]. The nucleotide site is a spacious cavity located at the interface of C_A_ and C_B_. In particular, several residues of switch B participate in substrate binding, and one of the main effects of CaM is to stabilize this switch in its active conformation [176]. 

The crystal structure of EF-CaM in complex with 3′-deoxy-ATP, PDB 1xfv [177] was used for docking of ATP and CTP (Figure 5B, C). According to the position of two Mg^2+^ ions, one coordinated with D491, D493 and H577, the other with the α-, β- and γ-phosphates of the substrates, two-metal-ion catalysis has been suggested [177]. Nucleophilic attack of the deprotonated 3′-oxygen on the α-phosphorus is the first step. Molecular dynamics simulations revealed structural conditions facilitating this attack [177]: a 3′-endo conformation of the ribosyl moiety, direct coordination of the 3′-oxygen by the metal, and activation of a water molecule by H351 leading to deprotonization of the 3′-OH group. Alternatively, one-metal-ion catalysis is possible as well. In previous crystal structures of EF-ACD complexed with CaM and 3′-deoxy-ATP [176], only one ytterbium ion is present in place of the "upper" magnesium ion in Figure 5, and H351 is in a position where it can directly deprotonate the 3′-OH group. However, H351 may be substituted by lysine without significant loss of adenylyl cyclase activity and without change of the pH optimum [177]. Therefore, H351 probably does not act as catalytic base, but rather stabilizes an OH^−^ ion near the 3′-OH group or deprotonates a neutral water molecule. 

In the triphosphate and the ribosyl regions, the binding modes of ATP and CTP are obviously not different. Coordination with Mg^2+^ leads to cyclic folding of the triphosphate groups. Additional salt bridges and/or charge-assisted hydrogen bonds with K346, K353, K372 and the backbone NH of S354 are formed by the β- and the γ-phosphates. The α- and β-phosphate groups are involved in charge-assisted hydrogen bonds with R329. A water molecule connects the α-phosphate with E588. The ribosyl moieties in 3′-endo conformations contact the side chains of L348 and H577 and form electrostatic interactions between the ring oxygens and the amide NH_2_ of N583 as well as between the 3′-oxygen atoms and both Mg^2+^ ions. The anthraniloyl groups of (M)ANT nucleotides protrude from the catalytic site. Hydrophobic interactions with F586 mostly account for their generally higher affinity compared to the parent nucleotides [158].

Both nucleotide bases occupy the same pocket mainly consisting of amino acids of switch B and align their ring planes with the side chain of N583. The amino groups of adenine and cytosine are involved in two hydrogen bonds with the backbone oxygens of T548 and T579. In the case of adenine, a third hydrogen bond of the nitrogen in 1-position with the backbone NH of T548 is formed. The main reason for the low affinity of UTP, MANT-UTP and MANT-GTP (Table 1 and Table 2) is that these hydrogen bonds are impossible with guanine and uracil as bases. The greater van der Waals surface (by ~22 Å^2^) and number of hydrogen bonds of ATP would rather indicate lower binding affinity of CTP. However, in the presence of Mn^2+^, the K_m_ value of CTP is ~3 times lower than that of ATP (Table 2), and MANT-CTP is a ~6 times more potent EF inhibitor than its ATP analog (Table 1). Possibly a water molecule contributes to CTP binding: Figure 5C shows that it may be placed in an ideal position, forming four hydrogen bonds which bridge the cytosine oxygen with the side chains of R329 and E580.

According to the related binding modes and similar K_m_ values, it is somewhat surprising that CTP (and UTP) are less efficient substrates of EF compared to ATP. The k_cat_ values of ATP are 35- to 100-fold greater than those of CTP. Tentative molecular dynamics simulations have shown that pyrimidine nucleotides tend to leave their original docking position due to high flexibility of the enzyme-substrate complex. Thus, alternative non-catalytic binding modes may be possible in the case of CTP (and UTP). In contrast, the adenine base of ATP (van der Waals surface ~22 Å greater than that of cytosine, additional direct hydrogen bond) is more tightly bound to switch A and B, resulting in a certain degree of immobilization of the enzyme-substrate complex and thus in more efficient catalysis.

Surprisingly, the type of the metal cation mainly affects affinity of substrates and inhibitory MANT-nucleotides (K_m_, K_i_ with Mn^2+^ < K_m_, K_i_ with Mg^2+^). Since the binding constants of UTP and PP_i_, respectively, for Mg^2+^ and Mn^2+^ are very similar [179], different direct interactions of the substrates or the products with the cations can be excluded as main reason. Also optimal Mg^2+^- and Mn^2+^-oxygen distances in some enzymes are similar (mean distances for coordination nr. 6: Mg^2+^-O, 2.07 Å, Mn^2+^-O, 2.17Å). However, the coordination sphere of Mn^2+^ is somewhat more flexible in comparison to Mg^2+^, and the dissociation of carboxylate ions and water is facilitated [180]. It may be thus suggested that higher nucleotide affinity with Mn^2+^ as cofactor is mainly due to greater flexibility and better reorganisation of the active sites of Mn^2+^ enzymes, leading to faster association kinetics.

## 5. Potential Cellular Targets of Novel Cyclic Nucleotides: Protein Kinases, Phosphodiesterases and Cyclic Nucleotide-Gated Ion Channels

The natural occurrence of cCMP, a novel cyclic nucleotide second messenger, was discussed controversially about 30 years ago, and was later supposedly confirmed by mass spectrometry methods [181]. However, the previously used mass spectrometry method was not sufficiently sensitive to discriminate the tentative cCMP from other molecules exhibiting similar molecular mass. In nine organs of rat, tentative cytidylyl cyclase (CC) activities were inversely proportional to the age of the animals [182,183]. Assumed cCMP levels in urine obtained from leukemic patients were very much elevated in comparison to normal human samples as determined by radioimmunoassay methods [184]. The existence of cUMP and cIMP has also been suggested to occur in mammalian cells [185]. Interestingly, cCMP was found to be an important modulator of immune responses. In macrophages, the cell permeant cCMP-analog dibutyryl-cCMP inhibited thromboxane B_2_ and leukotriene B_4_ formation [186]. In human neutrophils, dibutyryl-cCMP inhibited superoxide radical formation and the rise in cytosolic Ca^2+^ induced by a chemotactic peptide, resulting in neutrophil inactivation [187]. Taken together, these findings point to cCMP being a potential novel second messenger and a possible point of attack for bacterial exotoxins, although the existence of cCMP in mammalian cells has still to be confirmed. Using a very sensitive and specific mass spectrometry method, a preliminary congress report indicates that, indeed, cCMP does occur physiologically [188].

EF has been shown to be powerful in cycling CTP, UTP and ITP to form cCMP, cUMP and cIMP, respectively [163]. Additionally, EF effects independent from the cAMP/PKA pathway have been demonstrated [127,171]. Therefore, the question poses which could be possible targets for EF-induced intracellular accumulation of novel cyclic nucleotides.

### 5.1. Protein Kinases (PKs)

The PKs constitute one of the largest gene families encoded by the human genome and play a key regulatory role in many cellular pathways including cell division, cell death, growth, differentiation, and memory [11,189]. Protein kinase A (PKA) and protein kinase G (PKG) are the best characterized targets of cAMP and cGMP, respectively [11]. PKA is comprised of two types of subunits. Two catalytic subunits bind to a regulatory subunit dimer to form an inactive holoenzyme complex. Binding of cAMP to the regulatory subunit causes the catalytic subunit to be unleashed allowing it to phosphorylate its protein substrates. These substrates include membranous ion channels, key metabolic enzymes in the cytoplasm and transcription factors in the nucleus that regulate gene transcription. The regulatory domains of PKA exist in several isoforms, *i.e.*, RIα, RIIα, RIβ and RIIβ, differing in tissue distribution and sub-cellular distribution [190,191].

As the main effector of the nitric oxide/cGMP signaling cascade, cGMP-dependent protein kinase (PKG) regulates smooth muscle tone, inhibits platelet activation, and modulates neuronal functions [192]. In mammalian cells, two different genes encode a soluble type I PKG and a membrane-anchored type II PKG. PKG I comprises two splice variants (α and β) that differ in the first ∼100 amino acids, resulting in unique dimerization and autoinhibitory domains. PKG-I and PKG-II consist of homodimers of two identical subunits composed of a regulatory domain and a catalytic domain. Binding of cGMP to the two allosterically interacting cGMP-binding sites of the regulatory domain activates PKG activity.

Desch *et al.* have demonstrated relaxation of murine aorta as well as inhibition of platelet aggregation via activation of PKGIα and PKGIβ by the membrane-permeable cCMP analog dibutyryl-cCMP [193]. Both, cCMP-induced vascular relaxation and inhibition of platelet aggregation were absent in PKGI deficiency. Using radiochemical *in vitro* methods for assessing PK activities, Wolter *et al.* have investigated the activation of PKA and PKG by multiple cNMPs [194]. In agreement with the literature, cGMP was the most potent and effective activator of PKGIα. However, the nucleotide binding site of PKGIα also accommodated multiple other cyclic nucleotides as cAMP, cCMP, cIMP, cUMP and cXMP also activated the enzyme with cAMP and cCMP activating PKGIα with an efficacy amounting to ~55% of that of cGMP. The different maximum effects of cyclic nucleotides on PKG activation may result from differential hydrogen bonding of various nucleotides with the kinase allowing unique active PKG conformations comparable to the concept of ligand-specific receptor conformations in the field of G protein-coupled receptors. Consequently, cGMP may affect full PKG agonism while other cyclic nucleotides behave as partial PKG agonists. This partial agonism of cCMP on PKGIα may not represent a contradiction to cCMPs high effectivity at inducing vascular relaxation and inhibition of platelet aggregation as cCMP may not be subject to degradation by PDEs (see below). Therefore, EF-induced cCMP accumulation yielding high concentrations and cCMP-induced PKG effects are conceivable. Ultimate proof for such a full/partial agonism model of PKG activation will have to be obtained in crystallization studies of PKG with various nucleotides. 

All cyclic nucleotides studied by Wolter *et al.* [194] activated PKA with cAMP being the most potent activator for both, RIα and RIIα. RIα was activated in the order of potency cAMP > cIMP > cGMP > cCMP > cUMP > cXMP and with similar efficacy. Cyclic nucleotides activated RIIα in the order of potency cAMP > cIMP > cGMP > cUMP > cCMP > cXMP and with about similar efficacy. Interestingly, the activation profile of RIα by cAMP in the presence of 10 µM cCMP yielded a reduction of the pEC_50_ and a lower Hill slope. cCMP is a low-potency but high-efficacy activator of PKA RIα and RIIα. It is therefore conceivable that exogenously applied cCMP may elicit biological effects via PKG and PKA. The higher potency of cCMP to PKG as compared to PKA may explain the much larger contribution of PKG to cCMP-induced vascular relaxation. 

The work by Wolter *et al.* [194] is in good agreement with the literature. By incubating rat liver homogenate with cCMP, ATP as a phosphate donor and a histone substrate followed by positive-ion fast-atom bombardment mass spectrometric analysis of the enzyme incubation mixture, protein kinase activity specifically responsive to cCMP has been found [195]. Moreover, ten proteins were identified in brain tissue undergoing phosphorylation due to challenge with cCMP [196,197]. In the study described by Ding *et al.*, murine brain homogenates were incubated for a series of time periods with constant amounts of ATP together with one of three different cyclic nucleotides, cAMP, cGMP or cCMP, or ATP alone as a “blank” control experiment [197]. Phosphorylated proteins in cCMP-incubated mouse brain homogenate were characterized using an online IMAC-nano-LC/MS platform for phosphopeptide profiling [197]. Several cCMP-challenged phosphoproteins were identified, including formin homology 2 domain-containing protein 1, a member of a diverse family of proteins that have been shown to interact with Rho-family GTPases in order to organize the cytoskeleton of the cell and also regulate gene expression. MAP-kinase activating death domain isoform 8 and one particular protein kinase were identified as being unique to the cCMP-containing incubation and are both involved in apoptosis. Most recently, RIα and RIIα of PKA were identified as cCMP-binding proteins using cCMP-modified agarose matrices [198]. Taken together, these data render protein kinases conceivable target structures affected by EF-induced cNMPs.

### 5.2. Cyclic Nucleotide Phosphodiesterases (PDEs)

PDEs degrading cyclic nucleotide second messengers thus limiting cellular signaling in terms of time and space are essential regulators of various signaling pathways [17]. The PDE superfamily is divided into eleven families depending on substrate specificity, regulatory properties and cellular function. Clinically, isoform-specific PDE inhibitors modulating cAMP and cGMP degradation are of great importance, e.g., in the treatment of erectile dysfunction and pulmonary arterial hypertension. Recently, Reinecke *et al.* have investigated if purified human PDEs bind and hydrolyze other cyclic nucleotides besides cAMP and cGMP [199]. Following *in vitro* incubation of various cyclic nucleotides with distinct PDE isoforms, the reaction mixtures were subject to HPLC-MS/MS analysis in order to monitor potential reaction products. With a lower limit of quantitation of < 100 nM for both, various cyclic nucleotides as well as the corresponding nucleoside 5′-monophosphates being the reaction products, a highly sensitive quantitation method was established. In agreement with the literature, it was found that PDEs 3A, 3B and 4B hydrolyzed cAMP more effectively than cGMP, PDE8A was cAMP-selective, PDEs 1B and 5A showed the expected preference for cGMP, and PDE2A cleaved cAMP and cGMP about equally well. Strikingly, the PDEs studied exhibited much broader substrate specificity than described in the literature so far [200,201]. PDE1B did not only hydrolyze cAMP and cGMP but also cIMP and cXMP at high rates [199]. PDE2 hydrolyzed cIMP at similar rates as cAMP and cGMP. PDE9A, PDE5A and the two isoforms of PDE3 were capable of degrading any tested cyclic nucleotide, except for cCMP. The cyclic pyrimidine nucleotides cUMP and cTMP were accepted as substrates by several PDEs including PDE3A, PDE3B, PDE5A and PDE9A, whereas no PDE was capable of hydrolyzing cCMP under the experimental conditions described. Therefore, Reinecke *et al.* extended the incubation time up to 24 h in order to exclude the presence of a very low cCMP-degrading PDE activity in the enzyme preparations [199]. Under these extreme conditions, cyclic nucleotide hydrolysis by PDEs increased for cAMP, cGMP and cUMP, unless complete turnover was already observed after one hour. In marked contrast, even with the extremely extended incubation time, cCMP was not degraded at all. If cCMP cannot be degraded by mammalian PDEs, EF-induced formation of cCMP in host immune cells could cause substantial cCMP accumulation, resulting in toxic cellular effects.

In marked contrast to the study described by Reinecke *et al.*, previous studies postulated the existence of a cCMP-degrading PDE [202,203,204]. Newton *et al*. incubated cCMP together with rat tissue homogenate followed by positive-ion fast-atom bombardment mass spectrometric analysis of the incubation mixture after termination of the reaction [204]. It remains to be clarified whether a known PDE, not studied so far by Reinecke *et al.* [199] accounts for the previously claimed cCMP-degrading PDE activity.

### 5.3. Cyclic Nucleotide-Gated Ion Channels (CNGs)

CNGs are integral membrane proteins regulating ion fluxes across membranes thereby controlling membrane excitability [164,165,166]. To regulate ion fluxes, channels adapt open or closed conformations by undergoing conformational changes, a process which is allosterically regulated by changes in membrane voltage and binding of cyclic nucleotides. cNMPs bind directly to a cytoplasmic binding domain on the channel which, in turn, is allosterically coupled to the opening of the pore in these channels [205]. Due to their four sites for cooperative binding, their low affinities for cyclic nucleotides and a lack of desensitization in the presence of cyclic nucleotides, CNG channels represent fast and sensitive molecular switches. The intracellular carboxyl terminal domain of CNG channels contains a highly conserved stretch of amino acids that forms the binding site for cyclic nucleotides. This region has significant sequence similarity to the cyclic nucleotide binding domains of other cyclic nucleotide binding proteins, including cGMP- and cAMP-dependent protein kinases [165,166,206]. Interestingly, CNG channel conformations are not exclusively regulated by cAMP and cGMP. On rod CNG channels, cGMP behaves as a full agonist, and moreover, cAMP and cIMP are partial agonists, with cIMP being even more efficient than cAMP [164,207,208,209]. Intriguingly, a recent congress report revealed that cCMP is a partial activator of CNG channels [210], reminiscent to the partial PKG activation by cCMP [194]. As EF is capable of forming cCMP and cIMP, it is conceivable that besides the well-known, traditional cNMPs, novel EF-induced cNMPs might also work as ligands on CNGs to exert deleterious manipulations of cell biology. The ability of EF-induced formation of cNMPs to manipulate CNG function remains to be elucidated.

## 6. Unresolved Questions and Future Studies

Future studies will clarify the effect of bacterial adenylyl cyclase toxins on cNMP pools in intact cells and changes in gene expression patterns induced by specific cNMPs will be characterized. Most importantly, research will focus on the identification of all target proteins of novel cNMPs, on their role in the progression of anthrax pathogenesis as well as on the development of future treatment options. The capability of the mammalian PDE and MRP systems to degrade and extrude cNMPs will be assessed (Figure 3B). Furthermore, the role of potential NTP depletion processes due to the excessively high enzymatic EF activities will be investigated. In a more general view on cellular signaling, the question poses if cCMP, cUMP and cIMP could constitute novel endogenous signaling molecules. The existence of cCMP and cUMP in mammalian cells has recently been conclusively demonstrated by mass spectrometry methods [188]. In order to qualify as second messenger, an intracellular molecule has to fulfill several criteria. (i) The second messenger is produced following stimulation by a first messenger (hormone, neurotransmitter, local mediator). (ii) The second messenger elicits specific cellular effects. (iii) The actions of the second messenger are terminated by specific inactivation mechanisms. (iv) The effects of the second messenger can be mimicked by cell membrane-permeable second messenger molecules. (v) The effect of the second messenger is mimicked by the action of a bacterial or plant toxin. The former four are obligatory, the latter criterion is optional, depending on the availability of appropriate experimental probes. For the formation of cCMP, cUMP and cIMP, there currently is no known first messenger. The question poses which enzymes are capable of producing novel cNMPs found in mammalian tissue, adenylyl cyclases, guanylyl cyclases or unknown nucleotidyl cyclases. Mammalian soluble guanylyl cyclase (sGC) was recently shown to from cAMP, cCMP, cIMP and cUMP in addition to cGMP [211]. Considering the high conformational flexibility of mACs and the fact that MANT-CTP is equally potent at inhibiting mACs 1 and 5 and EF, it is well possible that ACs 1 and 5 also produce, to some extent, cCMP. Perhaps, further specific mammalian nucleotidyl cyclases exist. The cellular effects of cCMP are just beginning to be understood (Figure 3B). Most importantly, the cell membrane-permeable analog of cCMP, dibutyryl-cCMP, inhibits chemotactic peptide-stimulated superoxide anion formation in human neutrophils [187], which is compatible with the host defense-compromising function of EF [23,28]. In addition, dibutyryl-cCMP elicits relaxation of vascular smooth muscle [193]. cCMP-induced vasodilatation could contribute to EF-induced edema formation, the name-giving biological effect of the toxin, and to EF-induced septic shock, a severe pathophysiological condition involving vasodilatation as well [23,28]. For cUMP and cIMP, inactivation mechanisms by PDE have been shown [199]. PDE activity specific for cCMP has been described in mammalian tissue homogenate [203], but has not yet been confirmed in recombinant PDE systems [199]. Thus, our knowledge on novel cNMPs as potential second messengers is still very incomplete, but the unequivocal demonstration of nucleotidyl cyclase activity of bacterial “AC” toxins has opened the door for exciting novel avenues of research.

## 7. Conclusions

The *Bacillus anthracis* exotoxin EF clearly possesses the potential to form cCMP, cUMP and cIMP in addition to cAMP [163]. Similar to EF, the adenylyl cyclase toxin CyaA secreted by *Bordetella pertussis*, the causative agent of whooping cough, was recently shown to form cCMP, cIMP and cUMP, too [163]. Therefore, the formation of novel cyclic nucleotides impairing host defense may be a more common strategy used by many bacterial exotoxins. It is well conceivable that the cAMP-independent toxic EF effects described in the literature [127,171] may result from yet unknown manipulations of cellular signaling mechanisms by accumulation of cCMP, cUMP and cIMP, respectively. Hence the challenge remains to identify the cellular target proteins of novel cyclic nucleotides. Future studies will investigate PKs, PDEs, CNGs as well as MRPs as potential targets of novel, EF-induced cyclic nucleotides clearing the way to innovative treatment strategies.
